# Supplementary material for: Isolating, identifying and evaluating of oil degradation strains for the air-assisted microbial enhanced oil recovery process
Source: PLoS One. 2021 Jan 25;16(1):e0243976. doi: 10.1371/journal.pone.0243976 (PMC7834137; doi:10.1371/journal.pone.0243976)
Supplement: S1 Data — (DOCX) [file pone.0243976.s001.docx]

| time/d | microbial population of 3.0mg/L | the dissolved oxygen of 3.0mg/L | microbial population of 4.5mg/L | the dissolved oxygen of 4.5mg/L | microbial population of 5.5mg/L | the dissolved oxygen of 5.5mg/L |
| --- | --- | --- | --- | --- | --- | --- |
| 0 | 0.06 | 4.5 | 0.05 | 5.5 | 0.04 | 6.5 |
| 0.08 | 0.06 | 3.8 | 0.06 | 4.91 | 0.04 | 6.02 |
| 0.17 | 0.07 | 3.1 | 0.06 | 4.32 | 0.05 | 5.58 |
| 0.25 | 1.32 | 2.62 | 1.2 | 3.9 | 0.78 | 5.14 |
| 0.33 | 2.88 | 2.2 | 2.6 | 3.55 | 1.66 | 4.96 |
| 0.42 | 7.25 | 1.95 | 5.14 | 3 | 2.82 | 4.5 |
| 0.5 | 14.5 | 1.47 | 12 | 2.63 | 5.62 | 3.89 |
| 0.58 | 22.9 | 1.35 | 21.5 | 2.18 | 11.2 | 3.62 |
| 0.67 | 30.5 | 1.21 | 30.1 | 1.64 | 19.5 | 3.3 |
| 0.75 | 31.3 | 1.13 | 30.8 | 1.35 | 22.4 | 3.08 |
| 0.83 | 32.1 | 1.05 | 31 | 1.19 | 25.1 | 2.87 |
| 0.92 | 32.4 | 0.92 | 31.3 | 1.07 | 25.1 | 2.59 |
| 1 | 33.1 | 0.81 | 31.5 | 0.96 | 25.1 | 2.34 |
| 1.25 | 33.1 | 0.79 | 31.7 | 0.91 | 25.7 | 2.28 |
| 1.5 | 33.1 | 0.75 | 31.8 | 0.88 | 25.7 | 2.16 |
| 1.92 | 33.1 | 0.72 | 31.8 | 0.83 | 25.7 | 2.04 |
| 2.42 | 30.4 | 0.68 | 29.6 | 0.8 | 24.6 | 1.98 |
| 2.92 | 29.1 | 0.65 | 28.5 | 0.78 | 23.4 | 1.95 |
| 3.42 | 28.3 | 0.65 | 27.4 | 0.78 | 22.6 | 1.95 |
| 3.92 | 27.9 | 0.65 | 27 | 0.78 | 21.3 | 1.95 |
| 4.42 | 27.3 | 0.65 | 26.5 | 0.78 | 20.5 | 1.95 |
| 4.92 | 26.8 | 0.65 | 26.2 | 0.78 | 19.8 | 1.95 |
| 5.5 | 26.8 | 0.65 | 26.2 | 0.78 | 19.8 | 1.95 |

| time/d | the dissolved oxygen of 3.0mg/L | the dissolved oxygen of 4.5mg/L | the dissolved oxygen of 5.5mg/L |
| --- | --- | --- | --- |
| 0 | 3 | 4.5 | 5.5 |
| 0.08 | 2.5 | 3.8 | 4.91 |
| 0.17 | 2.2 | 3.1 | 4.32 |
| 0.25 | 1.8 | 2.62 | 3.9 |
| 0.33 | 1.6 | 2.2 | 3.55 |
| 0.42 | 1.3 | 1.95 | 3 |
| 0.5 | 1.1 | 1.47 | 2.63 |
| 0.58 | 1 | 1.35 | 2.18 |
| 0.67 | 0.9 | 1.21 | 1.64 |
| 0.75 | 0.8 | 1.13 | 1.35 |
| 0.83 | 0.7 | 1.05 | 1.19 |
| 0.92 | 0.65 | 0.92 | 1.07 |
| 1 | 0.64 | 0.81 | 0.96 |
| 1.25 | 0.6 | 0.79 | 0.91 |
| 1.5 | 0.55 | 0.75 | 0.88 |
| 1.92 | 0.54 | 0.72 | 0.83 |
| 2.42 | 0.53 | 0.68 | 0.8 |
| 2.92 | 0.52 | 0.65 | 0.78 |
| 3.42 | 0.51 | 0.65 | 0.77 |
| 3.92 | 0.51 | 0.64 | 0.76 |
| 4.42 | 0.5 | 0.63 | 0.76 |
| 4.92 | 0.5 | 0.63 | 0.76 |
| 5.5 | 0.5 | 0.63 | 0.76 |

|  | control group | 5.5 | 0.5 |
| --- | --- | --- | --- |
| C5 | 0.0226 | 0.2415 | 0.2415 |
| C6 | 0.2228 | 0.16748 | 0.16748 |
| C7 | 0.3828 | 0.37584 | 0.37584 |
| C8 | 0.4193 | 0.25498 | 0.25498 |
| C9 | 0.5523 | 0.33203 | 0.33203 |
| C10 | 0.5915 | 0.32451 | 0.32451 |
| C11 | 0.5683 | 0.0715 | 0.0383 |
| C12 | 0.723 | 1.028 | 0.1331 |
| C13 | 1.1162 | 4.1409 | 0.9452 |
| C14 | 1.3145 | 6.0014 | 2.3011 |
| C15 | 2.0303 | 7.2072 | 3.7359 |
| C16 | 2.0364 | 7.2787 | 4.353 |
| C17 | 2.5011 | 6.4073 | 4.4874 |
| C18 | 2.7168 | 5.9588 | 4.7836 |
| C19 | 2.9857 | 4.7661 | 3.855 |
| C20 | 3.2044 | 4.1361 | 3.7392 |
| C21 | 3.5346 | 3.7916 | 3.3742 |
| C22 | 3.5241 | 3.2952 | 4.0836 |
| C23 | 3.6633 | 2.8221 | 4.0502 |
| C24 | 3.6472 | 2.3171 | 3.8081 |
| C25 | 3.9248 | 1.9484 | 3.267 |
| C26 | 3.8944 | 1.6407 | 2.6252 |
| C27 | 4.201 | 1.5145 | 1.4412 |
| C28 | 4.3183 | 1.4015 | 1.2651 |
| C29 | 2.7166 | 1.6025 | 1.7214 |
| C30 | 7.3868 | 1.4108 | 2.6192 |
| C31 | 4.7796 | 1.1457 | 2.0385 |
| C32 | 3.7862 | 0.6763 | 3.0979 |
| C33 | 3.896 | 0.358 | 0.264 |
| C34 | 3.508 | 0.2534 | 0.27 |
| C35 | 3.2841 | 0.2228 | 0.173 |
| C36 | 2.9696 | 0.2635 | 0.0708 |
| C37 | 2.846 | 0.4118 | 0.1175 |
| C38 | 2.8502 | 0.6478 | 0.196 |
| C39 | 2.3862 | 0.8388 | 0.0366 |
| C40 | 2.3462 | 1.1838 | 0.4095 |

| pv | Vi | Voi | Vwi | oil recovery | water cut | △P | |
| --- | --- | --- | --- | --- | --- | --- | --- |
| 1.09579 | 14.3 | 2.93 | 11.37 | 0.22452 | 0.9 | | 47 |
| 1.18774 | 15.5 | 2.95 | 12.55 | 0.22605 | 0.98333 | | 47 |
| 1.22605 | 16 | 2.98 | 13.02 | 0.22835 | 0.94 | | 47 |
| 1.28736 | 16.8 | 3 | 13.8 | 0.22989 | 0.975 | | 47 |
| 1.341 | 17.5 | 3.01 | 14.49 | 0.23065 | 0.98571 | | 47 |
| 1.39464 | 18.2 | 3.02 | 15.18 | 0.23142 | 0.98571 | | 47 |
| 1.47893 | 19.3 | 3.03 | 16.27 | 0.23218 | 0.99091 | | 47 |
| 1.49425 | 19.5 | 3.04 | 16.46 | 0.23295 | 0.95 | | 47 |
| 1.53257 | 20 | 3.05 | 16.95 | 0.23372 | 0.98 | | 47 |
| 1.57088 | 20.5 | 3.05 | 17.45 | 0.23372 | 1 | | 47 |
| 1.6092 | 21 | 3.05 | 17.95 | 0.23372 | 1 | | 47 |
| 1.66284 | 21.7 | 3.05 | 18.65 | 0.23372 | 1 | | 47 |
| 1.75479 | 22.9 | 3.05 | 19.85 | 0.23372 | 1 | | 47 |
| 1.83142 | 23.9 | 3.05 | 20.85 | 0.23372 | 1 | | 47 |
| 1.89272 | 24.7 | 3.05 | 21.65 | 0.23372 | 1 | | 47 |
| 1.9387 | 25.3 | 3.05 | 22.25 | 0.23372 | 1 | | 47 |
| 2.02299 | 26.4 | 3.05 | 23.35 | 0.23372 | 1 | | 47 |
| 2.11494 | 27.6 | 3.05 | 24.55 | 0.23372 | 1 | | 47 |
| 2.16858 | 28.3 | 3.05 | 25.25 | 0.23372 | 1 | | 47 |
| 2.24521 | 29.3 | 3.05 | 26.25 | 0.23372 | 1 | | 47 |
| 2.61303 | 34.1 | 3.05 | 31.05 | 0.23372 | 1 | | 47 |
| 2.79693 | 36.5 | 3.05 | 33.45 | 0.23372 | 1 | | 47 |

| pv | Vi | Voi | Vwi | oil recovery | water cut | △P |
| --- | --- | --- | --- | --- | --- | --- |
| 1.78845 | 35 | 6.1 | 28.9 | 0.3117 | 0.94 | 28 |
| 2.24834 | 44 | 6.2 | 37.8 | 0.31681 | 0.935 | 25 |
| 2.5907 | 50.7 | 6.2 | 44.5 | 0.31681 | 1 | 25 |
| 2.8513 | 55.8 | 6.2 | 49.6 | 0.31681 | 1 | 25 |
| 3.163 | 61.9 | 6.2 | 55.7 | 0.31681 | 1 | 25 |
| 3.45938 | 67.7 | 6.2 | 61.5 | 0.31681 | 1 | 25 |
| 3.56157 | 69.7 | 6.2 | 63.5 | 0.31681 | 1 | 25 |
| 3.61267 | 70.7 | 6.2 | 64.5 | 0.31681 | 1 | 25 |
| 3.66377 | 71.7 | 6.2 | 65.5 | 0.31681 | 1 | 25 |
| 3.8324 | 75 | 6.2 | 68.8 | 0.31681 | 1 | 25 |
| 3.93459 | 77 | 6.2 | 70.8 | 0.31681 | 1 | 25 |
| 4.08789 | 80 | 6.2 | 73.8 | 0.31681 | 1 | 25 |
| 4.34338 | 85 | 6.2 | 78.8 | 0.31681 | 1 | 25 |

| pv | Vi | Voi | Vwi | oil recovery | water cut | △P |
| --- | --- | --- | --- | --- | --- | --- |
| 0 | 0 | 0 | 0 | 0 | 0 | 0 |
| 0 | 0 | 0 | 0 | 0 | 0 | 0 |
| 0 | 0 | 0 | 0 | 0 | 0 | 0 |
| 0.02498 | 0.3 | 0.3 | 0 | 0.02498 | 0 | 0 |
| 0.04996 | 0.6 | 0.5 | 0.1 | 0.04163 | 0.33333 | 17 |
| 0.07494 | 0.9 | 0.68 | 0.22 | 0.05662 | 0.4 | 24 |
| 0.09159 | 1.1 | 0.8 | 0.3 | 0.06661 | 0.4 | 27 |
| 0.10824 | 1.3 | 0.89 | 0.41 | 0.0741 | 0.55 | 28 |
| 0.1249 | 1.5 | 0.97 | 0.53 | 0.08077 | 0.6 | 29 |
| 0.14155 | 1.7 | 1.04 | 0.66 | 0.08659 | 0.65 | 30 |
| 0.16653 | 2 | 1.11 | 0.89 | 0.09242 | 0.76667 | 31 |
| 0.25812 | 3.1 | 1.3 | 1.8 | 0.10824 | 0.82727 | 32 |
| 0.2831 | 3.4 | 1.3 | 2.1 | 0.10824 | 1 | 30 |
| 0.29975 | 3.6 | 1.35 | 2.25 | 0.11241 | 0.75 | 29 |
| 0.32473 | 3.9 | 1.4 | 2.5 | 0.11657 | 0.83333 | 28 |
| 0.35803 | 4.3 | 1.45 | 2.85 | 0.12073 | 0.875 | 27 |
| 0.39134 | 4.7 | 1.5 | 3.2 | 0.1249 | 0.875 | 25 |
| 0.42465 | 5.1 | 1.55 | 3.55 | 0.12906 | 0.875 | 38 |
| 0.42881 | 5.15 | 1.56 | 3.59 | 0.12989 | 0.8 | 39 |
| 0.43297 | 5.2 | 1.565 | 3.635 | 0.13031 | 0.9 | 40 |
| 0.43714 | 5.25 | 1.57 | 3.68 | 0.13072 | 0.9 | 42 |
| 0.4413 | 5.3 | 1.575 | 3.725 | 0.13114 | 0.9 | 42 |
| 0.44546 | 5.35 | 1.58 | 3.77 | 0.13156 | 0.9 | 42 |
| 0.44963 | 5.4 | 1.58 | 3.82 | 0.13156 | 1 | 42 |
| 0.45379 | 5.45 | 1.58 | 3.87 | 0.13156 | 1 | 42 |
| 0.45795 | 5.5 | 1.58 | 3.92 | 0.13156 | 1 | 42 |
| 0.46211 | 5.55 | 1.58 | 3.97 | 0.13156 | 1 | 39 |
| 0.46628 | 5.6 | 1.59 | 4.01 | 0.13239 | 0.8 | 34 |
| 0.4746 | 5.7 | 1.6 | 4.1 | 0.13322 | 0.9 | 24 |
| 0.48293 | 5.8 | 1.625 | 4.175 | 0.1353 | 0.75 | 25 |
| 0.49126 | 5.9 | 1.64 | 4.26 | 0.13655 | 0.85 | 27 |
| 0.49958 | 6 | 1.66 | 4.34 | 0.13822 | 0.8 | 29 |
| 0.50791 | 6.1 | 1.68 | 4.42 | 0.13988 | 0.8 | 31 |
| 0.51124 | 6.14 | 1.685 | 4.455 | 0.1403 | 0.875 | 31 |
| 0.51624 | 6.2 | 1.69 | 4.51 | 0.14072 | 0.91667 | 31 |
| 0.52456 | 6.3 | 1.7 | 4.6 | 0.14155 | 0.9 | 29 |
| 0.54122 | 6.5 | 1.73 | 4.77 | 0.14405 | 0.85 | 27 |
| 0.54954 | 6.6 | 1.74 | 4.86 | 0.14488 | 0.9 | 25 |
| 0.56619 | 6.8 | 1.75 | 5.05 | 0.14571 | 0.95 | 23 |
| 0.5995 | 7.2 | 1.8 | 5.4 | 0.14988 | 0.875 | 28 |
| 0.62448 | 7.5 | 1.84 | 5.66 | 0.15321 | 0.86667 | 29 |
| 0.64946 | 7.8 | 1.88 | 5.92 | 0.15654 | 0.86667 | 32 |
| 0.70774 | 8.5 | 1.9 | 6.6 | 0.1582 | 0.97143 | 28 |
| 0.7577 | 9.1 | 2 | 7.1 | 0.16653 | 0.83333 | 24 |
| 0.79933 | 9.6 | 2.1 | 7.5 | 0.17485 | 0.8 | 25 |
| 0.89925 | 10.8 | 2.3 | 8.5 | 0.19151 | 0.83333 | 26 |
| 0.94921 | 11.4 | 2.4 | 9 | 0.19983 | 0.83333 | 26 |
| 0.99917 | 12 | 2.5 | 9.5 | 0.20816 | 0.83333 | 24 |
| 1.0408 | 12.5 | 2.6 | 9.9 | 0.21649 | 0.8 | 21 |
| 1.08243 | 13 | 2.7 | 10.3 | 0.22481 | 0.8 | 22 |
| 1.11574 | 13.4 | 2.8 | 10.6 | 0.23314 | 0.75 | 23 |
| 1.1657 | 14 | 2.98 | 11.02 | 0.24813 | 0.7 | 24 |
| 1.19067 | 14.3 | 3.05 | 11.25 | 0.25396 | 0.76667 | 23 |
| 1.29059 | 15.5 | 3.25 | 12.25 | 0.27061 | 0.83333 | 22 |
| 1.33222 | 16 | 3.4 | 12.6 | 0.2831 | 0.7 | 21 |
| 1.39883 | 16.8 | 3.5 | 13.3 | 0.29142 | 0.875 | 20 |
| 1.45712 | 17.5 | 3.58 | 13.92 | 0.29808 | 0.88571 | 20 |
| 1.5154 | 18.2 | 3.7 | 14.5 | 0.30808 | 0.82857 | 20 |
| 1.60699 | 19.3 | 3.8 | 15.5 | 0.3164 | 0.90909 | 20 |
| 1.64863 | 19.8 | 3.85 | 15.95 | 0.32057 | 0.9 | 20 |
| 1.68193 | 20.2 | 3.9 | 16.3 | 0.32473 | 0.875 | 20 |
| 1.70691 | 20.5 | 3.92 | 16.58 | 0.32639 | 0.93333 | 20 |
| 1.74854 | 21 | 3.95 | 17.05 | 0.32889 | 0.94 | 20 |
| 1.80683 | 21.7 | 3.98 | 17.72 | 0.33139 | 0.95714 | 20 |
| 1.90674 | 22.9 | 4 | 18.9 | 0.33306 | 0.98333 | 20 |
| 1.99001 | 23.9 | 4.05 | 19.85 | 0.33722 | 0.95 | 20 |
| 2.05662 | 24.7 | 4.08 | 20.62 | 0.33972 | 0.9625 | 20 |
| 2.10658 | 25.3 | 3.2 | 22.1 | 0.26644 | 0.91667 | 42 |
| 2.19817 | 26.4 | 3.25 | 23.15 | 0.27061 | 0.95455 | 42 |
| 2.29808 | 27.6 | 3.3 | 24.3 | 0.27477 | 0.95833 | 42 |
| 2.35637 | 28.3 | 3.35 | 24.95 | 0.27893 | 0.92857 | 42 |
| 2.43963 | 29.3 | 3.4 | 25.9 | 0.2831 | 0.95 | 42 |
| 2.8393 | 34.1 | 3.7 | 30.4 | 0.30808 | 0.9375 | 42 |
| 3.03913 | 36.5 | 3.9 | 32.6 | 0.32473 | 0.91667 | 42 |
| 3.37219 | 40.5 | 4.1 | 36.4 | 0.34138 | 0.95 | 42 |
| 3.78851 | 45.5 | 4.2 | 41.3 | 0.34971 | 0.98 | 42 |
| 4.62115 | 55.5 | 4.3 | 51.2 | 0.35803 | 0.99 | 42 |
| 5.204 | 62.5 | 4.4 | 58.1 | 0.36636 | 0.98571 | 42 |
| 5.6453 | 67.8 | 4.4 | 63.4 | 0.36636 | 1 | 39 |
| 6.06162 | 72.8 | 4.4 | 68.4 | 0.36636 | 1 | 39 |

| pv | Vi | Voi | Vwi | oil recovery | water cut | △P |
| --- | --- | --- | --- | --- | --- | --- |
| 0 | 0 | 0 | 0 | 0 | 0 | 0 |
| 0 | 0 | 0 | 0 | 0 | 0 | 0 |
| 0 | 0 | 0 | 0 | 0 | 0 | 0 |
| 0.01785 | 0.3 | 0.3 | 0 | 0.01785 | 0 | 0 |
| 0.05354 | 0.9 | 0.9 | 0 | 0.05354 | 0 | 19 |
| 0.05948 | 1 | 1 | 0 | 0.05948 | 0 | 24 |
| 0.07733 | 1.3 | 1.3 | 0 | 0.07733 | 0 | 31 |
| 0.11302 | 1.9 | 1.9 | 0 | 0.11302 | 0 | 32 |
| 0.12492 | 2.1 | 2.1 | 0 | 0.12492 | 0 | 34 |
| 0.14276 | 2.4 | 2.3 | 0.1 | 0.13682 | 0.33333 | 38 |
| 0.16656 | 2.8 | 2.5 | 0.3 | 0.14871 | 0.5 | 35 |
| 0.19035 | 3.2 | 2.65 | 0.55 | 0.15763 | 0.625 | 34 |
| 0.21415 | 3.6 | 2.8 | 0.8 | 0.16656 | 0.625 | 33 |
| 0.23794 | 4 | 2.9 | 1.1 | 0.17251 | 0.75 | 32 |
| 0.27958 | 4.7 | 3.06 | 1.64 | 0.18202 | 0.77143 | 31 |
| 0.35691 | 6 | 3.2 | 2.8 | 0.19035 | 0.89231 | 28 |
| 0.38665 | 6.5 | 3.28 | 3.22 | 0.19511 | 0.84 | 24 |
| 0.41639 | 7 | 3.35 | 3.65 | 0.19927 | 0.86 | 21 |
| 0.47588 | 8 | 3.45 | 4.55 | 0.20522 | 0.9 | 19 |
| 0.53536 | 9 | 3.55 | 5.45 | 0.21117 | 0.9 | 28 |
| 0.59485 | 10 | 3.6 | 6.4 | 0.21415 | 0.95 | 29 |
| 0.69002 | 11.6 | 3.7 | 7.9 | 0.22009 | 0.9375 | 27 |
| 0.70192 | 11.8 | 3.73 | 8.07 | 0.22188 | 0.85 | 20 |
| 0.73166 | 12.3 | 3.8 | 8.5 | 0.22604 | 0.86 | 19 |
| 0.7733 | 13 | 3.9 | 9.1 | 0.23199 | 0.85714 | 20 |
| 0.80305 | 13.5 | 4 | 9.5 | 0.23794 | 0.8 | 26 |
| 0.83279 | 14 | 4.1 | 9.9 | 0.24389 | 0.8 | 28 |
| 0.86253 | 14.5 | 4.2 | 10.3 | 0.24984 | 0.8 | 30 |
| 0.89227 | 15 | 4.28 | 10.72 | 0.2546 | 0.84 | 27 |
| 0.92202 | 15.5 | 4.32 | 11.18 | 0.25697 | 0.92 | 24 |
| 0.95176 | 16 | 4.35 | 11.65 | 0.25876 | 0.94 | 22 |
| 0.9815 | 16.5 | 4.38 | 12.12 | 0.26054 | 0.94 | 20 |
| 1.01124 | 17 | 4.4 | 12.6 | 0.26173 | 0.96 | 19 |
| 1.14211 | 19.2 | 4.5 | 14.7 | 0.26768 | 0.95455 | 15 |
| 1.21944 | 20.5 | 4.6 | 15.9 | 0.27363 | 0.92308 | 17 |
| 1.30272 | 21.9 | 4.8 | 17.1 | 0.28553 | 0.85714 | 18 |
| 1.33841 | 22.5 | 4.9 | 17.6 | 0.29148 | 0.83333 | 19 |
| 1.36815 | 23 | 5 | 18 | 0.29742 | 0.8 | 20 |
| 1.42764 | 24 | 5.1 | 18.9 | 0.30337 | 0.9 | 20 |
| 1.49307 | 25.1 | 5.2 | 19.9 | 0.30932 | 0.90476 | 19 |
| 1.5704 | 26.4 | 5.4 | 21 | 0.32122 | 0.84615 | 20 |
| 1.70127 | 28.6 | 5.6 | 23 | 0.33312 | 0.90909 | 21 |
| 1.89162 | 31.8 | 5.9 | 25.9 | 0.35096 | 0.90625 | 22 |
| 2.1474 | 36.1 | 6 | 30.1 | 0.35691 | 0.97674 | 17 |
| 2.37345 | 39.9 | 6.2 | 33.7 | 0.36881 | 0.94737 | 15 |
| 2.61733 | 44 | 6.4 | 37.6 | 0.3807 | 0.95122 | 16 |
| 3.01588 | 50.7 | 6.6 | 44.1 | 0.3926 | 0.97015 | 17 |
| 3.31926 | 55.8 | 6.7 | 49.1 | 0.39855 | 0.98039 | 18 |
| 3.68211 | 61.9 | 6.8 | 55.1 | 0.4045 | 0.98214 | 18 |
| 4.02713 | 67.7 | 6.8 | 60.9 | 0.4045 | 1 | 17 |
| 4.14609 | 69.7 | 6.8 | 62.9 | 0.4045 | 1 | 16 |
| 4.20558 | 70.7 | 6.8 | 63.9 | 0.4045 | 1 | 15 |
| 4.26506 | 71.7 | 6.8 | 64.9 | 0.4045 | 1 | 15 |
| 4.35429 | 73.2 | 6.8 | 66.4 | 0.4045 | 1 | 15 |
| 4.68741 | 78.8 | 6.8 | 72 | 0.4045 | 1 | 15 |
| 5.01457 | 84.3 | 6.8 | 77.5 | 0.4045 | 1 | 15 |
| 5.32984 | 89.6 | 6.8 | 82.8 | 0.4045 | 1 | 15 |
| 5.65701 | 95.1 | 6.8 | 88.3 | 0.4045 | 1 | 15 |
| 5.97228 | 100.4 | 6.8 | 93.6 | 0.4045 | 1 | 15 |

| Swd | krw水 | kro-2 | uw/uo | fw | fw' | kro of water flooding | kro-1 | krw-1 | Sw1 |
| --- | --- | --- | --- | --- | --- | --- | --- | --- | --- |
| 0.02743 | 0 | 0.91642 | 0.41047 | 0 | 0.2029 | 1 | 0.91217 | 4.65E-05 | 0.378 |
| 0.05552 | 1.00E-03 | 0.83588 | 0.41047 | 0.00284 | 0.40169 | 0.85 | 0.82722 | 2.77E-04 | 0.392 |
| 0.1097 | 0.005 | 0.69442 | 0.41047 | 0.01647 | 0.61711 | 0.65 | 0.67735 | 0.00155 | 0.419 |
| 0.15585 | 0.009 | 0.58757 | 0.41047 | 0.0337 | 1.00034 | 0.5 | 0.56372 | 0.00377 | 0.442 |
| 0.22007 | 0.016 | 0.45837 | 0.41047 | 0.07149 | 1.83377 | 0.38 | 0.42612 | 0.00903 | 0.474 |
| 0.27224 | 0.023 | 0.36884 | 0.41047 | 0.14005 | 2.12667 | 0.29 | 0.33103 | 0.01547 | 0.5 |
| 0.31238 | 0.03 | 0.30869 | 0.41047 | 0.16931 | 1.9487 | 0.225 | 0.26755 | 0.02191 | 0.52 |
| 0.35653 | 0.035 | 0.25065 | 0.41047 | 0.2219 | 2.29179 | 0.15 | 0.20694 | 0.03061 | 0.542 |
| 0.39265 | 0.049 | 0.20909 | 0.41047 | 0.26099 | 6.75686 | 0.11 | 0.16419 | 0.04908 | 0.56 |
| 0.44683 | 0.09 | 0.15594 | 0.41047 | 0.52596 | 8.6633 | 0.075 | 0.11091 | 0.08027 | 0.587 |
| 0.47291 | 0.11 | 0.13401 | 0.41047 | 0.60752 | 6.10937 | 0.06 | 0.08964 | 0.09779 | 0.6 |
| 0.51706 | 0.159 | 0.10183 | 0.41047 | 0.73979 | 5.04335 | 0.048 | 0.05968 | 0.13565 | 0.622 |
| 0.5572 | 0.2 | 0.07756 | 0.41047 | 0.81934 | 4.39184 | 0.03 | 0.03857 | 0.16526 | 0.642 |
| 0.56522 | 0.229 | 0.07323 | 0.41047 | 0.84519 | 3.01031 | 0.027 | 0.035 | 0.18339 | 0.65 |
| 0.59934 | 0.252 | 0.05667 | 0.41047 | 0.88255 | 2.32348 | 0.022 | 0.0221 | 0.20317 | 0.663 |
| 0.61539 | 0.28 | 0.04984 | 0.41047 | 0.90328 | 1.791 | 0.021 | 0.01723 | 0.22196 | 0.671 |
| 0.65151 | 0.3 | 0.03657 | 0.41047 | 0.92912 | 1.78363 | 0.015 | 0.00881 | 0.24689 | 0.682 |
| 0.65552 | 0.32 | 0.03527 | 0.41047 | 0.93895 | 1.10922 | 0.008 | 0.00808 | 0.26841 | 0.691 |
| 0.73379 | 0.42 | 0.01571 | 0.41047 | 0.9746 | 0.91405 | 0 | 4.60E-04 | 0.3628 | 0.749 |

|  | water cat | recovery |  | water cat | recovery |
| --- | --- | --- | --- | --- | --- |
| 0 | 0 | 0 | 0 | 0 | 0 |
| 0.103 | 0 | 8.9 | 1.00E-04 | 0.81986 | 0.01685 |
| 0.179 | 0 | 18.8 | 5.00E-04 | 0.85186 | 0.05605 |
| 0.308 | 17.7 | 28.7 | 0.00145 | 0.90179 | 0.14707 |
| 0.369 | 23.1 | 32.7 | 0.01664 | 1.2315 | 1.68433 |
| 0.436 | 53.9 | 36.6 | 0.03328 | 1.39997 | 3.36926 |
| 0.795 | 71 | 46.6 | 0.04992 | 1.52733 | 5.05417 |
| 0.821 | 90 | 47.1 | 0.09985 | 1.81208 | 10.10889 |
| 0.897 | 95 | 47.6 | 0.11649 | 1.87456 | 11.79381 |
| 1 | 96.5 | 48.1 | 0.13313 | 1.92819 | 13.47873 |
| 1.333 | 98 | 48.5 | 0.21633 | 2.17494 | 21.90338 |
| 1.513 | 87.5 | 50.7 | 0.23297 | 2.22564 | 23.58832 |
| 1.744 | 99 | 51 | 0.24961 | 2.30806 | 25.27325 |
| 1.923 | 98 | 51.5 | 0.26625 | 2.45489 | 26.95817 |
| 2.103 | 97 | 52.5 | 0.2829 | 2.79931 | 28.64307 |
| 2.59 | 99 | 53.06 | 0.29954 | 3.67975 | 30.32774 |
| 3.282 | 100 | 53.06 | 0.31618 | 6.09569 | 32.01231 |
| 4 | 100 | 53.06 | 0.33282 | 12.56988 | 33.69709 |
| 4 |  |  | 0.34946 | 25.87248 | 35.38202 |
| 4 |  |  | 0.3661 | 48.67441 | 37.06724 |
| 4 |  |  | 0.38274 | 66.12599 | 38.24824 |
| 4 |  |  | 0.39938 | 73.81654 | 39.12933 |
| 4 |  |  | 0.4493 | 82.6927 | 41.02033 |
| 4 |  |  | 0.46595 | 84.17734 | 41.51701 |
| 4 |  |  | 0.51587 | 86.96819 | 42.7907 |
| 4 |  |  | 0.53251 | 87.67276 | 43.16012 |
| 4 |  |  | 0.56579 | 88.89835 | 43.83573 |
| 4 |  |  | 0.59907 | 89.97591 | 44.43859 |
| 4 |  |  | 0.61571 | 90.50011 | 44.71479 |
| 4 |  |  | 0.64899 | 91.50925 | 45.22009 |
| 4 |  |  | 0.66564 | 91.95558 | 45.45204 |
| 4 |  |  | 0.70395 | 92.80189 | 45.92848 |
| 4 |  |  | 0.78987 | 93.10908 | 46.79778 |
| 4 |  |  | 0.83204 | 94.63903 | 47.18587 |
| 4 |  |  | 0.9757 | 95.65867 | 48.25294 |
| 4 |  |  | 0.99845 | 95.81136 | 48.41579 |
| 4 |  |  | 1.16486 | 96.1897 | 49.49298 |
| 4 |  |  | 1.33144 | 96.30038 | 50.50232 |
| 4 |  |  | 1.33183 | 95.30641 | 50.50773 |
| 4 |  |  | 1.34791 | 93.42286 | 50.72997 |
| 4 |  |  | 1.36455 | 92.53672 | 50.95595 |
| 4 |  |  | 1.38119 | 91.66825 | 51.17841 |
| 4 |  |  | 1.39784 | 91.82223 | 51.39677 |
| 4 |  |  | 1.41448 | 91.00452 | 51.61025 |
| 4 |  |  | 1.43112 | 91.22133 | 51.81795 |
| 4 |  |  | 1.44776 | 91.47141 | 52.01897 |
| 4 |  |  | 1.4644 | 92.24811 | 52.2126 |
| 4 |  |  | 1.48104 | 92.73465 | 52.39858 |
| 4 |  |  | 1.49768 | 93.31881 | 52.57698 |
| 4 |  |  | 1.52667 | 93.76041 | 52.86724 |
| 4 |  |  | 1.56962 | 94.6399 | 53.26418 |
| 4 |  |  | 1.58088 | 95.04705 | 53.36636 |
| 4 |  |  | 1.62551 | 95.71853 | 53.74449 |
| 4 |  |  | 1.66409 | 96.35347 | 54.05072 |
| 4 |  |  | 1.71969 | 97.03965 | 54.43971 |
| 4 |  |  | 1.74729 | 97.79619 | 54.61706 |
| 4 |  |  | 1.74746 | 98.45337 | 54.61741 |
| 4 |  |  | 1.74785 | 98.45287 | 54.61837 |
| 4 |  |  | 1.74875 | 98.45441 | 54.62061 |
| 4 |  |  | 1.75086 | 98.46257 | 54.6258 |
| 4 |  |  | 1.75577 | 98.49731 | 54.6376 |
| 4 |  |  | 1.76393 | 98.5859 | 54.65607 |
| 4 |  |  | 1.78281 | 98.76198 | 54.69346 |
| 4 |  |  | 1.79722 | 98.86645 | 54.71958 |
| 4 |  |  | 1.8305 | 99.08358 | 54.76837 |
| 4 |  |  | 1.86378 | 99.16956 | 54.81258 |
| 4 |  |  | 1.89706 | 99.19098 | 54.85565 |
| 4 |  |  | 1.9137 | 99.19707 | 54.87702 |
| 4 |  |  | 1.94698 | 99.20366 | 54.91942 |
| 4 |  |  | 1.98027 | 99.20833 | 54.96158 |
| 4 |  |  | 2.04683 | 99.2161 | 55.04525 |
| 4 |  |  | 2.08011 | 99.21992 | 55.08679 |
| 4 |  |  | 2.14668 | 99.22785 | 55.16923 |
| 4 |  |  | 2.17996 | 99.23193 | 55.21012 |
| 4 |  |  | 2.21324 | 99.23618 | 55.25079 |
| 4 |  |  | 2.24652 | 99.2406 | 55.29123 |
| 4 |  |  | 2.2798 | 99.24507 | 55.33142 |
| 4 |  |  | 2.31308 | 99.24974 | 55.37137 |
| 4 |  |  | 2.34637 | 99.25461 | 55.41106 |
| 4 |  |  | 2.37965 | 99.25958 | 55.45048 |
| 4 |  |  | 2.41293 | 99.26472 | 55.48963 |
| 4 |  |  | 2.49028 | 99.27722 | 55.57907 |
| 4 |  |  | 2.57934 | 99.29226 | 55.67991 |
| 4 |  |  | 2.66254 | 99.30694 | 55.77216 |
| 4 |  |  | 2.74575 | 99.3222 | 55.86238 |
| 4 |  |  | 2.84559 | 99.3415 | 55.96757 |
| 4 |  |  | 2.86223 | 99.34471 | 55.98501 |
| 4 |  |  | 3.07857 | 99.38991 | 56.19614 |
| 4 |  |  | 3.24497 | 99.42538 | 56.34911 |
| 4 |  |  | 3.41138 | 99.46112 | 56.49257 |
| 4 |  |  | 3.7944 | 99.54063 | 56.77396 |
| 4 |  |  | 4.01046 | 99.58318 | 56.91802 |

| Reservoir radius | air injection of 3000m3/d | air injection of 4000m3/d | air injection of 5000m3/d | safe oxygen concentration corresponding to 3000m3/d | safe oxygen concentration corresponding to 4000m4/d | safe oxygen concentration corresponding to 5000m5/d |
| --- | --- | --- | --- | --- | --- | --- |
| 0 | 2864.35 | 3820.35 | 4776.35 | 1434 | 1912 | 2390 |
| 50 | 2642.169 | 3598.169 | 4554.169 | 1434 | 1912 | 2390 |
| 100 | 1975.627 | 2931.627 | 3887.627 | 1434 | 1912 | 2390 |
| 150 | 864.7228 | 1820.723 | 2776.723 | 1434 | 1912 | 2390 |
| 200 | -690.543 | 265.4572 | 1221.457 | 1434 | 1912 | 2390 |

| Reservoir radius | water injection of 30m3/d | water injection of 50m4/d | water injection of 70m5/d | safe oxygen concentration corresponding to 30m3/d | safe oxygen concentration corresponding to50m4/d | safe oxygen concentration corresponding to70m5/d |
| --- | --- | --- | --- | --- | --- | --- |
| 0 | 6369.683 | 3820.35 | 2727.779 | 3186 | 1912 | 1365 |
| 50 | 6147.503 | 3598.169 | 2505.598 | 3186 | 1912 | 1365 |
| 100 | 5480.96 | 2931.627 | 1839.055 | 3186 | 1912 | 1365 |
| 150 | 4370.056 | 1820.723 | 728.1514 | 3186 | 1912 | 1365 |
| 200 | 2814.791 | 265.4572 | -827.114 | 3186 | 1912 | 1365 |

| date | recovery | date of water flooding | recovery of water flooding |
| --- | --- | --- | --- |
| 1990 | 0 | 1990 | 0 |
| 1990 | 1.05E-04 | 1990 | 1.05E-04 |
| 1990 | 3.48E-04 | 1990 | 3.48E-04 |
| 1990 | 9.14E-04 | 1990 | 9.14E-04 |
| 1990.001 | 0.00222 | 1990.001 | 0.00222 |
| 1990.001 | 0.00523 | 1990.001 | 0.00523 |
| 1990.003 | 0.01201 | 1990.003 | 0.01201 |
| 1990.007 | 0.02686 | 1990.007 | 0.02686 |
| 1990.015 | 0.05809 | 1990.015 | 0.05809 |
| 1990.032 | 0.1202 | 1990.032 | 0.1202 |
| 1990.064 | 0.23576 | 1990.064 | 0.23576 |
| 1990.123 | 0.43699 | 1990.123 | 0.43699 |
| 1990.222 | 0.7745 | 1990.222 | 0.7745 |
| 1990.381 | 1.32351 | 1990.381 | 1.32351 |
| 1990.496 | 1.72047 | 1990.496 | 1.72047 |
| 1990.691 | 2.36363 | 1990.691 | 2.36363 |
| 1990.751 | 2.56163 | 1990.751 | 2.56163 |
| 1990.81 | 2.75869 | 1990.81 | 2.75869 |
| 1990.872 | 2.96409 | 1990.872 | 2.96409 |
| 1990.968 | 3.27961 | 1990.968 | 3.27961 |
| 1991 | 3.38718 | 1991 | 3.38718 |
| 1991.102 | 3.72368 | 1991.102 | 3.72368 |
| 1991.25 | 4.21526 | 1991.25 | 4.21526 |
| 1991.457 | 4.90031 | 1991.457 | 4.90031 |
| 1991.729 | 5.79159 | 1991.729 | 5.79159 |
| 1992 | 6.66503 | 1992 | 6.66503 |
| 1992.363 | 7.81981 | 1992.363 | 7.81981 |
| 1992.814 | 9.23171 | 1992.814 | 9.23171 |
| 1993.003 | 9.81992 | 1993.003 | 9.81992 |
| 1993.309 | 10.75736 | 1993.309 | 10.75736 |
| 1993.704 | 11.95444 | 1993.704 | 11.95444 |
| 1994.003 | 12.84404 | 1994.003 | 12.84404 |
| 1994.393 | 13.98621 | 1994.393 | 13.98621 |
| 1994.915 | 15.49026 | 1994.915 | 15.49026 |
| 1995.003 | 15.74244 | 1995.003 | 15.74244 |
| 1995.574 | 17.35405 | 1995.574 | 17.35405 |
| 1996.003 | 18.55856 | 1996.003 | 18.55856 |
| 1996.554 | 20.08217 | 1996.554 | 20.08217 |
| 1997.005 | 21.32482 | 1997.005 | 21.32482 |
| 1997.552 | 22.80387 | 1997.552 | 22.80387 |
| 1998.005 | 24.01325 | 1998.005 | 24.01325 |
| 1998.456 | 25.20196 | 1998.456 | 25.20196 |
| 1999.005 | 26.62432 | 1999.005 | 26.62432 |
| 1999.489 | 27.85203 | 1999.489 | 27.85203 |
| 2000.005 | 29.13003 | 2000.005 | 29.13003 |
| 2000.594 | 30.5369 | 2000.594 | 30.5369 |
| 2001.326 | 32.19576 | 2001.008 | 31.49869 |
| 2002.008 | 33.64919 | 2001.614 | 32.83789 |
| 2002.99 | 35.54762 | 2002.008 | 33.68728 |
| 2004.008 | 37.29966 | 2002.644 | 34.95562 |
| 2005.369 | 39.28426 | 2003.008 | 35.65366 |
| 2006.011 | 40.15194 | 2003.599 | 36.71075 |
| 2006.593 | 40.87338 | 2004.008 | 37.40709 |
| 2007.056 | 41.41151 | 2004.686 | 38.4666 |
| 2007.331 | 41.72256 | 2005.011 | 38.96275 |
| 2007.806 | 42.22105 | 2005.618 | 39.80035 |
| 2008.011 | 42.43238 | 2006.011 | 40.31448 |
| 2008.534 | 42.93472 | 2006.578 | 41.01261 |
| 2009.225 | 43.5452 | 2007.011 | 41.5065 |
| 2009.649 | 43.90711 | 2007.401 | 41.92991 |
| 2010.014 | 44.19887 | 2007.95 | 42.48384 |
| 2010.479 | 44.55378 | 2008.011 | 42.54473 |
| 2011.084 | 44.99426 | 2008.752 | 43.22114 |
| 2012.014 | 45.60031 | 2009.014 | 43.45353 |
| 2013.364 | 46.39995 | 2009.627 | 43.96105 |
| 2014.016 | 46.7642 | 2010.014 | 44.26942 |
| 2015.079 | 47.30219 | 2010.393 | 44.55587 |
| 2016.016 | 47.76152 | 2011.014 | 45.00394 |
| 2016.266 | 47.87963 | 2011.811 | 45.52714 |
| 2016.277 | 47.8898 | 2012.014 | 45.65725 |
| 2016.5 | 48.08597 | 2013.016 | 46.25107 |
| 2016.657 | 48.22342 | 2013.792 | 46.69388 |
| 2016.834 | 48.37753 | 2014.016 | 46.81602 |
| 2016.927 | 48.45741 | 2014.723 | 47.18113 |
| 2017.083 | 48.59024 | 2015.016 | 47.3303 |
| 2017.222 | 48.70783 | 2015.831 | 47.71857 |
| 2017.543 | 49.06834 | 2016.016 | 47.80593 |
| 2017.971 | 49.32238 | 2017.019 | 48.24881 |
| 2018.389 | 49.65434 | 2017.917 | 48.63174 |
| 2018.465 | 49.71363 | 2018.019 | 48.67429 |
| 2018.584 | 49.80366 | 2019.019 | 49.06865 |
| 2018.756 | 49.85924 | 2020.019 | 49.44711 |
| 2018.807 | 49.90592 | 2021.022 | 49.79722 |
| 2019.214 | 50.1394 | 2022.022 | 50.13497 |
| 2019.466 | 50.33246 | 2023.022 | 50.45666 |
| 2019.784 | 50.52316 | 2024.022 | 50.75496 |
| 2020.069 | 50.69488 | 2025.025 | 51.04522 |
| 2021.281 | 51.43013 | 2026.025 | 51.32007 |
| 2022.681 | 52.287 | 2027.025 | 51.57838 |
| 2023.613 | 52.84417 | 2028.025 | 51.82813 |
| 2024.081 | 53.11419 | 2029.027 | 52.06769 |
| 2024.547 | 53.376 | 2030.027 | 52.29653 |
| 2025.013 | 53.62985 | 2031.027 | 52.51543 |
| 2025.479 | 53.87574 | 2032.027 | 52.72491 |
| 2025.947 | 54.11441 | 2033.03 | 52.92594 |
| 2026.413 | 54.34477 | 2034.03 | 53.11795 |
| 2026.879 | 54.56777 | 2035.03 | 53.29844 |
| 2027.345 | 54.78374 | 2036.03 | 53.47417 |
| 2028.239 | 55.17415 | 2037.033 | 53.64119 |
| 2029.679 | 55.75013 | 2038.033 | 53.80135 |
| 2031.625 | 56.4487 | 2039.033 | 53.95549 |
| 2032.011 | 56.58404 | 2040.033 | 54.10398 |
| 2033.746 | 57.14133 | 2041.036 | 54.24765 |
| 2034.343 | 57.32715 | 2042.036 | 54.38595 |
| 2036.15 | 57.84336 | 2043.036 | 54.5196 |
| 2036.675 | 57.9898 | 2044.036 | 54.64886 |
| 2039.008 | 58.57801 | 2045.038 | 54.77432 |
| 2041.34 | 59.11446 | 2046.038 | 54.89502 |
| 2044.785 | 59.81722 | 2047.038 | 55.01268 |
| 2046.005 | 60.05591 | 2048.038 | 55.12731 |
| 2048.381 | 60.48719 | 2049.041 | 55.23896 |
| 2050.67 | 60.87556 | 2050.041 | 55.34699 |
| 2053.842 | 61.36991 | 2051.041 | 55.45192 |
| 2055.334 | 61.59388 | 2052.041 | 55.55396 |
| 2058.825 | 62.07735 | 2053.044 | 55.65259 |
| 2060 | 62.23562 | 2054.044 | 55.74869 |
| 2063.401 | 62.66108 | 2055.044 | 55.84219 |
| 2064.664 | 62.81483 | 2056.044 | 55.93315 |
| 2068.105 | 63.2068 | 2057.047 | 56.0219 |
| 2069.329 | 63.34305 | 2058.047 | 56.10753 |
| 2073.994 | 63.8217 | 2059.047 | 56.19107 |
| 2074.461 | 63.86937 | 2060.047 | 56.27274 |
| 2075.393 | 63.96316 | 2061.049 | 56.35287 |
| 2076.327 | 64.05558 | 2062.049 | 56.4312 |
| 2077.259 | 64.14641 | 2063.049 | 56.50756 |
| 2077.726 | 64.19129 | -- |  |
| 2078.193 | 64.23594 | -- |  |

| date | water cut | date of water flooding | water cut of water flooding |
| --- | --- | --- | --- |
| 1990 | 0 | 1990 | 0 |
| 1990 | 0.0043 | 1990 | 0.0043 |
| 1990 | 0.01006 | 1990 | 0.01006 |
| 1990 | 0.01773 | 1990 | 0.01773 |
| 1990.001 | 0.03009 | 1990.001 | 0.03009 |
| 1990.001 | 0.04986 | 1990.001 | 0.04986 |
| 1990.003 | 0.10573 | 1990.003 | 0.10573 |
| 1990.007 | 0.24532 | 1990.007 | 0.24532 |
| 1990.015 | 0.75551 | 1990.015 | 0.75551 |
| 1990.032 | 2.33716 | 1990.032 | 2.33716 |
| 1990.064 | 6.36735 | 1990.064 | 6.36735 |
| 1990.123 | 9.98833 | 1990.123 | 9.98833 |
| 1990.222 | 10.61983 | 1990.222 | 10.61983 |
| 1990.381 | 9.96082 | 1990.381 | 9.96082 |
| 1990.496 | 9.55774 | 1990.496 | 9.55774 |
| 1990.691 | 13.90884 | 1990.691 | 13.90884 |
| 1990.751 | 13.97139 | 1990.751 | 13.97139 |
| 1990.81 | 13.33922 | 1990.81 | 13.33922 |
| 1990.872 | 13.43218 | 1990.872 | 13.43218 |
| 1990.968 | 13.43077 | 1990.968 | 13.43077 |
| 1991 | 13.38518 | 1991 | 13.38518 |
| 1991.102 | 13.81053 | 1991.102 | 13.81053 |
| 1991.25 | 13.52337 | 1991.25 | 13.52337 |
| 1991.457 | 13.28248 | 1991.457 | 13.28248 |
| 1991.729 | 14.58024 | 1991.729 | 14.58024 |
| 1992 | 15.82174 | 1992 | 15.82174 |
| 1992.363 | 16.97612 | 1992.363 | 16.97612 |
| 1992.814 | 18.21689 | 1992.814 | 18.21689 |
| 1993.003 | 18.71297 | 1993.003 | 18.71297 |
| 1993.309 | 20.09197 | 1993.309 | 20.09197 |
| 1993.704 | 20.80671 | 1993.704 | 20.80671 |
| 1994.003 | 22.31459 | 1994.003 | 22.31459 |
| 1994.393 | 23.54872 | 1994.393 | 23.54872 |
| 1994.915 | 24.80865 | 1994.915 | 24.80865 |
| 1995.003 | 24.95523 | 1995.003 | 24.95523 |
| 1995.574 | 26.31113 | 1995.574 | 26.31113 |
| 1996.003 | 26.68483 | 1996.003 | 26.68483 |
| 1996.554 | 27.799 | 1996.554 | 27.799 |
| 1997.005 | 28.19481 | 1997.005 | 28.19481 |
| 1997.552 | 29.39834 | 1997.552 | 29.39834 |
| 1998.005 | 30.30069 | 1998.005 | 30.30069 |
| 1998.456 | 31.18259 | 1998.456 | 31.18259 |
| 1999.005 | 32.36191 | 1999.005 | 32.36191 |
| 1999.489 | 33.72757 | 1999.489 | 33.72757 |
| 2000.005 | 35.37255 | 2000.005 | 35.37255 |
| 2000.594 | 37.58233 | 2000.594 | 37.58233 |
| 2001.326 | 40.87057 | 2001.008 | 39.3853 |
| 2002.008 | 44.34459 | 2001.614 | 42.32296 |
| 2002.99 | 49.51697 | 2002.008 | 43.6824 |
| 2004.008 | 55.063 | 2002.644 | 47.90335 |
| 2005.369 | 61.9343 | 2003.008 | 49.98484 |
| 2006.011 | 64.68467 | 2003.599 | 53.2842 |
| 2006.593 | 67.62631 | 2004.008 | 55.56871 |
| 2007.056 | 69.66137 | 2004.686 | 59.16118 |
| 2007.331 | 70.45291 | 2005.011 | 60.19302 |
| 2007.806 | 72.60433 | 2005.618 | 63.99102 |
| 2008.011 | 73.07232 | 2006.011 | 65.80882 |
| 2008.534 | 74.91412 | 2006.578 | 67.8479 |
| 2009.225 | 76.93597 | 2007.011 | 70.2214 |
| 2009.649 | 77.69621 | 2007.401 | 71.65771 |
| 2010.014 | 79.13048 | 2007.95 | 73.66708 |
| 2010.479 | 80.06613 | 2008.011 | 73.79993 |
| 2011.084 | 80.98576 | 2008.752 | 76.16924 |
| 2012.014 | 82.9892 | 2009.014 | 76.78378 |
| 2013.364 | 84.5389 | 2009.627 | 78.39591 |
| 2014.016 | 85.41765 | 2010.014 | 79.16762 |
| 2015.079 | 86.77918 | 2010.393 | 80.29064 |
| 2016.016 | 87.20722 | 2011.014 | 81.14608 |
| 2016.266 | 87.63073 | 2011.811 | 82.87513 |
| 2016.277 | 86 | 2012.014 | 83.21105 |
| 2016.5 | 85.52456 | 2013.016 | 84.53706 |
| 2016.657 | 84 | 2013.792 | 85.101 |
| 2016.834 | 83.14259 | 2014.016 | 85.76175 |
| 2016.927 | 82.09135 | 2014.723 | 86.51375 |
| 2017.083 | 81 | 2015.016 | 86.71345 |
| 2017.222 | 80.36852 | 2015.831 | 87.55393 |
| 2017.543 | 80.9 | 2016.016 | 87.69637 |
| 2017.971 | 81.61821 | 2017.019 | 88.46489 |
| 2018.389 | 82.99695 | 2017.917 | 88.86892 |
| 2018.465 | 83.23976 | 2018.019 | 89.08279 |
| 2018.584 | 84 | 2019.019 | 89.70304 |
| 2018.756 | 84.8 | 2020.019 | 90.11826 |
| 2018.807 | 85.2 | 2021.022 | 90.88411 |
| 2019.214 | 85.8 | 2022.022 | 91.18172 |
| 2019.466 | 86.00296 | 2023.022 | 91.60046 |
| 2019.784 | 86.54836 | 2024.022 | 92.21114 |
| 2020.069 | 86.8255 | 2025.025 | 92.44211 |
| 2021.281 | 87.0365 | 2026.025 | 92.82321 |
| 2022.681 | 87.368 | 2027.025 | 93.25407 |
| 2023.613 | 87.79 | 2028.025 | 93.47886 |
| 2024.081 | 87.93462 | 2029.027 | 93.7621 |
| 2024.547 | 88.26962 | 2030.027 | 94.02454 |
| 2025.013 | 88.62652 | 2031.027 | 94.2846 |
| 2025.479 | 88.98296 | 2032.027 | 94.52998 |
| 2025.947 | 89.33564 | 2033.03 | 94.76505 |
| 2026.413 | 89.67859 | 2034.03 | 94.98637 |
| 2026.879 | 90.00845 | 2035.03 | 95.28728 |
| 2027.345 | 90.32343 | 2036.03 | 95.41146 |
| 2028.239 | 90.87665 | 2037.033 | 95.65108 |
| 2029.679 | 91.64339 | 2038.033 | 95.81799 |
| 2031.625 | 92.50553 | 2039.033 | 95.97556 |
| 2032.011 | 92.66247 | 2040.033 | 96.12257 |
| 2033.746 | 93.29356 | 2041.036 | 96.25908 |
| 2034.343 | 93.49312 | 2042.036 | 96.38885 |
| 2036.15 | 94.03288 | 2043.036 | 96.51054 |
| 2036.675 | 94.17673 | 2044.036 | 96.62488 |
| 2039.008 | 94.73485 | 2045.038 | 96.73302 |
| 2041.34 | 95.19557 | 2046.038 | 96.84808 |
| 2044.785 | 95.73891 | 2047.038 | 96.92729 |
| 2046.005 | 95.91183 | 2048.038 | 97.00652 |
| 2048.381 | 96.20881 | 2049.041 | 97.09238 |
| 2050.67 | 96.45698 | 2050.041 | 97.1788 |
| 2053.842 | 96.74509 | 2051.041 | 97.26002 |
| 2055.334 | 96.86455 | 2052.041 | 97.33521 |
| 2058.825 | 97.10738 | 2053.044 | 97.4317 |
| 2060 | 97.18541 | 2054.044 | 97.49004 |
| 2063.401 | 97.3876 | 2055.044 | 97.55833 |
| 2064.664 | 97.45647 | 2056.044 | 97.62431 |
| 2068.105 | 97.62104 | 2057.047 | 97.68848 |
| 2069.329 | 97.67519 | 2058.047 | 97.76382 |
| 2073.994 | 97.85655 | 2059.047 | 97.81838 |
| 2074.461 | 97.8692 | 2060.047 | 97.86774 |
| 2075.393 | 97.90717 | 2061.049 | 97.9128 |
| 2076.327 | 97.94061 | 2062.049 | 97.95437 |
| 2077.259 | 97.97307 | 2063.049 | 98.00612 |
| 2077.726 | 97.98895 | -- |  |
| 2078.193 | 98.00474 | -- |  |
