# Supplementary material for: Isolating, identifying and evaluating of oil degradation strains for the air-assisted microbial enhanced oil recovery process
Source: PLoS One. 2021 Jan 25;16(1):e0243976. doi: 10.1371/journal.pone.0243976 (PMC7834137; doi:10.1371/journal.pone.0243976)
Supplement: Attachment — Submitted filename: Response to Reviewers.docx [file pone.0243976.s002.docx]

Reviewer #1: I have read the paper with interest.

The manuscript topic is of interest for “PLOS ONE”.

The authors offer a work that potentially can improve our knowledge about this topic. I suggest that the authors consider a revision of their work along the following suggestions and questions.

1- It is suggested to discuss more about the findings of this study in the abstract.

Response: More about the findings of this study was discussed in the abstract. The experiment shows that water flooding, microbial flooding and air - assisted microbial flooding core flow experiments were carried out. Carbon distribution curve of biodegraded oil with different oxygen concentration was determined by chromatographic analysis. The long-chain alkanes are degraded by microorganisms. This process is very conducive to lowering the oil viscosity.

2- It is recommended to mention about the applications of this study at the end of the abstract.

The findings of this study can help for better understanding of …

Response: The applications of this study were mentioned about at the end of the abstract.

The findings of this study can help for better understanding of microbial enhanced oil recovery and improving the efficiency of microbial oil displacement.

3- I strongly recommend the authors to add one paragraph discussing the difference between their work and the previously performed studies in literature. In other words, what is the novelty of this work? I offer the authors to revise the abstract and introduction in order to incorporate the novelty of their work. This change motivates the readers of “PLOS ONE” to study this work with interest.

Response: The abstract and introduction were revised in order to incorporate the novelty of our work.

The microbial enhanced oil recovery faced with a problem that the inefficient reproduction of microorganisms in oxygen-deprived environments of the reservoir. To overcome this problem, a new type of air-assisted MEOR process was investigated.

4- The following relevant references can be cited in introduction where “At the same time. non-renewable resources, such as oil, are at risk of depletion, consequently, applying enhanced oil recovery (EOR) techniques in developed fields is crucial to the maximization of scarce oil resources.” is addressed:

• (2020), Experimental study on the viscosity behavior of silica nanofluids with different ions of electrolytes. Industrial & Engineering Chemistry Research, 59(8): 3575-3583.

• (2020), Interfacial energy for solutions of nanoparticles, surfactants, and electrolytes. American Institute of Chemical Engineers Journal, 66(4): e16891.

2019), A comprehensive review on interaction of nanoparticles with low salinity water and surfactant for enhanced oil recovery in sandstone and carbonate reservoirs. Fuel, 241: 1045-1057.

• (2019), Mathematical modelling of surface tension of nanoparticles in electrolyte solutions. Chemical Engineering Science, 197: 345-356.

Response: The following relevant references were cited in introduction.

5- It is suggested to add a figure in section 1 (Introduction) which shows the general sketch of the problem.

Response: A figure was added in section 1 (Introduction) to show the general sketch of this study.

6- It is recommended to discuss about the “Recent Theoretical, Field, and Experimental Studies” in the introduction.

Response: The introduction was modified, the “Recent Theoretical, Field, and Experimental Studies” was discussed in the introduction.

7- It is recommended to include a paragraph at the end of introduction to present the steps of the work like:

First, the materials and methods are presented. Then,…

Response: A paragraph at the end of introduction was added to present the steps of the work.

The steps of the work were as flows: First, the materials and methods are presented. Then, the strain was identified and the microbial oxygen consumption rate is determined. Then, the influence of dissolved oxygen concentration on the carbon number distribution of biodegraded oil is measured. Then, the physical simulation of air-assisted MEOR in heterogeneous porous media is carried out. Finally, the mathematical model of air-assisted MEOR is established, the air-assisted microbial flooding performance is predicted.

8- What are the advantages and disadvantages of this study? I recommend the authors to highlight this topic.

Response: The advantages and disadvantages of this study is low cost, powerful adaptability, simple construction, harmless reservoir and no-pollution. Furthermore, the air-assisted MEOR technology could overcome the inefficient reproduction of traditional MEOR in oxygen-deprived environments of the reservoir.

The disadvantage of this study is that continuous air injection into the reservoir comes with a risk of oxygen breakthrough. In such case, the oxygen concentration may be reduced to a safe level through microorganism growth.

9- It is recommended to keep the main governing equations in the text of manuscript and move the rest of equations to an appendix.

Response: The main governing equations in the study are reedited.

10- The title for the last section should be replaced by “Summary and Conclusions”.

The title for the last section has been replaced by “Summary and Conclusions”.

11- It is recommended to show the main remarks of this study in terms of bullets in last section (Summary and Conclusions).

Response: The main remarks of this study were shown in terms of bullets in last section (Summary and Conclusions).

12- It is suggested to add a nomenclature.

Response: The nomenclature in the study was rechecked and modified. Compared to traditional MEOR, The “air-assisted Microbial Enhanced Oil Recovery” is a new nomenclature.

Reviewer #2: The paper has investigated a new air-assited MEOR process. The paper proves a good addition in literature and add good addition in the field or EOR. Paper can be accepted after following revisions.

1. In the introduction part author has discussed about the background and concepts of MEOR process. The author should write in separate paragraphs, also add introduction about simulation part, and in the last paragraph discuss the summary of methodology in a bit more detail.

Add some of the recent articles about EOR, such as,

https://doi.org/10.1016/j.jclepro.2020.120777

<https://doi.org/10.1016/j.molliq.2020.113095>

Response: Introduction about simulation part was added, and in the last paragraph discuss the summary of methodology in a bit more detail. The recent articles about EOR were referenced.

2. Check for grammar and spelling mistakes.

Response: The grammar and spelling mistakes were checked and revised.

3. In section 2.1, “The cultures were stored at a temperature of 4 °C for later use” why this temperature was choosen? Discuss and provide reference if necessary.

Response: The cultures were stored at a temperature of 4 °C for later use, because the strain is dormant at this temperature. Therefore, 4°C is the optimum temperature for preserving the species.

4. If methodology in section 2.3 was adopted from some other research provide references, otherwise provide reasoning.

Response: The experiment was designed according to the definition of microbial oxygen consumption. Microbial oxygen consumption is the amount of dissolved oxygen in water consumed by microbial reproduction and metabolism. Oxygen consumption rate is the rate at which microorganisms use up oxygen for respiration. The experiment is referred to a paper published by Lei Guanglun in 2016.

5. Equation numbers should be in line, also provide references for all the equations which are used from some other research.

Response: Equation number was readjusted to be in line.

6. In table 3, the values should be in line.

Response: Table 3 has been reedited to be case - sensitive.

7. In Table 6, author has proposed different recovery rates because of different injection patterns, author has not discussed the effect of permeability, porosity and pressure difference on the recoevery rate. As these values are not constant, so author should take in account and discuss these parameters.

Response: Core flooding experiments were conducted using artificial cores, thus the parameters of artificial cores could not be exactly consistent. The permeability, porosity and pressure of artificial cores were as close as possible.

8. In Figure 9, captions and numbering should be placed properly.

Response: Figure 9 contains four diagrams:（a）is the core flooding experiment of water driving，(b) is the core flooding experiment of microbial flooding, (c) is the core flooding experiment of air- microorganisms-air- microorganisms flooding,(d)is the core flooding experiment of microorganisms-air- microorganisms-air flooding.

9. Check format for equations 14 and 15.

Response: Equations 14 and 15 have been reedited.
