# Supplementary material for: Isolating, identifying and evaluating of oil degradation strains for the air-assisted microbial enhanced oil recovery process
Source: PLoS One. 2021 Jan 25;16(1):e0243976. doi: 10.1371/journal.pone.0243976 (PMC7834137; doi:10.1371/journal.pone.0243976)
Supplement: Attachment — Submitted filename: Response to Reviewers-2.docx [file pone.0243976.s003.docx]

The authors have improved the manuscript "Separating and Evaluating Oil Degradation Strains for the Air-assisted Microbial Enhanced Oil Recovery Process" in somehow that is possible. However, there are some main points to be addressed before it can be proceeded further.

Lines 30-31: Incomplete statement. Check it: "The current rapid pace of economic and social development worldwide necessitates, ...."

Lines 30-31 was modified to “The current rapid pace of economic and social development worldwide necessitates the use of ever-increasing amounts of resources (Cheng et al., 2014). ”

Table 2: Determine how the parameters have analyzed? Which device or analysis is used to measure these parameters and elements?

The parameters have been analyzed by chemical titration.

Line 117: sterilization for 30 min in high-pressure steam. At what level of pressure? Clarify.

Line 117 was modified to “After sterilization for 30 min in high-pressure steam (0.12MPa), ”

Line 118: gas flow control devices. How many g-f devices are applied?

Line 117 was modified to “air was introduced into the first three flasks via a gas flow control devices to set the dissolved oxygen concentrations to the desired values of 3.0, 4.5, and 5.5 mg/L.”

What is/are difference(s) between Lines 116-120 and 134-137? After sterilization .....for 5 days.

“while stirring continuously, for 5 days” is bacterial culture process. Lines 116-120 were bacterial culture for determination of microbial oxygen consumption rate, Lines 134-137 were bacterial culture for Influence of dissolved oxygen concentration on the carbon number distribution.

Line 154: discuss more about "Buckley-Leverett theory".

Line 154 was modified to “Under the precondition of incompressible flow, the capillary pressure and gravity are not considered，the mathematical model used in this study is based on the Buckley-Leverett theory and Darcy’s law.”

Fi. 5: Did you adapt Fig. 4 from Cheng et al. or others? If yes, provide its Rightslink with a proper citation.

A sentence was added “Concrete method for oxygen-consumption model was shown in Fig.5( Cheng (Cheng et al., 2014).” before Fig.5.

Lines 270-271: "This strain has a strong ability to break down organics into molecules such as glycolipids and lipopeptides." Any reference?

Reference was add in Lines 270-271.

Fig.7: An elucidation is required here to state about the used digits in Fig. 7. Please provide a sufficient explanation about digits used in Fig. 7, maybe as Supplementary Data.

The digits represent the similarity of the strains to the database cultures.

Fig.8 (Right): It is not clear that the bars (y= -0.014x+0.702 ...) are related to which curve? Change color accordingly.

An indicator line indicates that the relationship of the bars (y= -0.014x+0.702 ...)and the curve.

Table 5 should be discussed more in the text. What are the results and their application in the process of MEOR?

Some sentences were added here. Table 5 shows that the oxygen consumption rate of microorganism is different with different oxygen content. This is because the metabolic rate of microorganisms was different under different oxygen content. The oxygen concentration distribution was calculated using the data of microbial metabolic rate.

Line 323: "As the table shows" which table?

This line was changed to “As Table 6 shows,”

Fig. 10 a: Why the trend of water cut is dis-order? Clarify within the text briefly.

Some sentences were added here. Although the water cut fluctuates in some moment, it shows a larger upward trend. The water cut curves of air-assisted microbial flooding have obvious fall during secondary water drive stage. It shows that the air-assisted microbial flooding is effective in water cut control during enhanced oil recovery process.

Remove below references from the list and also the text. They are irrelevant as recommended to the authors in the first stage:

Saheed O and Morteza D. Interfacial energy for solutions of nanoparticles, surfactants, and electrolytes[J]. American Institute of Chemical Engineers Journal, 2020, 66(4): e16891.

Saheed O and Morteza D. Experimental Study on the Viscosity Behavior of Silica Nanofluids with Different Ions of Electrolytes [J]. 2020, 59, 8, 3575–3583

The below references were remove from the list and also the text.
